# Effects of the Myokine Irisin on Stromal Cells from Swine Adipose Tissue

**DOI:** 10.3390/biom12121895

**Published:** 2022-12-17

**Authors:** Giuseppina Basini, Simona Bussolati, Stefano Grolli, Priscilla Berni, Rosanna Di Lecce, Francesca Grasselli

**Affiliations:** Dipartimento di Scienze Medico-Veterinarie, Università di Parma, Via del Taglio 10, 43126 Parma, Italy

**Keywords:** adipocytes, ASCs, oxidative stress

## Abstract

Irisin is a hormone able to reproduce some of the positive effects of physical activity and diet. Recently, we demonstrated the presence of Irisin at the ovarian level as a potential physiological regulator of follicular function. Adipose tissue is crucial for reproductive function through its metabolic activity and the production of adipokines. At present, the exact nature of adipocyte precursors is still under debate, but an important role has been assigned to the population of adipose tissue mesenchymal stromal cells (ASCs) of perivascular origin. It should be noted that, when appropriately stimulated, ASCs can differentiate into preadipocytes and, subsequently, adipocytes. Therefore, this present study was undertaken to explore the potential effect of Irisin on ASCs, known for their high differentiative potential. Since Irisin expression in ASCs was confirmed by PCR, we tested its potential effects on the main functional activities of these cells, including proliferation (BrdU uptake); metabolic activity (ATP production); redox status, evaluated as the generation of free molecules such as superoxide anion and nitric oxide; and scavenger activities, assessed as both enzymatic (superoxide dismutase) and non-enzymatic antioxidant power. Moreover, we tested the effect of Irisin on ASCs adipogenic differentiation. BrdU uptake was significantly (*p* < 0.001) inhibited by Irisin, while ATP production was significantly (*p* < 0.05) increased. Both superoxide anion and nitric oxide generation were significantly increased (*p* < 0.001) by Irisin, while scavenger activity was significantly reduced (*p* < 0.05). Irisin was found to significantly (*p* < 0.05) inhibit ASCs adipogenic differentiation. Taken together, the present results suggest a potential local role of Irisin in the regulation of adipose tissue function.

## 1. Introduction

Irisin, which was discovered by Bostrom et al. [1] and named after Iris, the messenger of the Gods, is a cytokine mainly secreted by skeletal muscle in response to exercise. It is therefore categorized as a myokine [2]. It is well known that a lack of physical activity and a sedentary lifestyle expose an individual to several metabolic, cardiovascular and neurological diseases. Over the past decade, Irisin has been demonstrated to mimic exercise’s positive effects, and therapeutic application has been suggested even if several molecular details are still lacking [3]. In a previous study, we demonstrated the potential role of Irisin in the physiological function of the swine ovarian follicle, thus suggesting its involvement in the regulation of the reproductive process [4], mainly exerted through its metabolic activity and the production of adipokines. The pivotal role played by this cytokine could also be represented by its control of the local immune system homeostasis, for example, regulating the functional state of macrophages. Recently, Yang et al. [5] suggested that peri-ovarian fat contributes to paracrine control of the adjacent ovarian tissue, playing a role in oocyte maturation. Growth factors and adipokine are secreted by mature adipocytes, whose activity is, in turn, dependent on proper tissue homeostasis. Protein secretion by adipocytes has been recently documented as well as its involvement in the “browning” of these cells [6]. It should be noted that a crucial hallmark of reproductive function refers to the endocrine function of adipose tissue. Although there is no absolute certainty about the exact nature of adipocyte precursors, an important role is assigned to the population of adipose tissue mesenchymal stromal cells (ASCs) of perivascular origin. When appropriately stimulated, ASCs can differentiate into preadipocytes and, subsequently, adipocytes [7,8,9]. These models have been mainly studied in vitro, but strong indications support the hypothesis that they also apply in vivo [9]. Therefore, this present research was undertaken to explore the potential local expression and function of the myokine in swine ASCs, isolated accordingly to our previous studies [10,11]. In particular, the effects on proliferation, metabolic activity, and redox status were assessed. In addition, the potential role of Irisin in ASCs adipogenic differentiation was examined.

## 2. Materials and Methods

All reagents were obtained from Sigma (St. Louis, MO, USA) unless otherwise specified. 

### 2.1. Isolation of Adipose Stromal Cells (ASCs) from Swine Abdominal Subcutaneous Adipose Tissue

The adipose tissue was collected from mature gilts (*n* = 5; Large White × Landrace) at 7–8 months of age, weighing 160–180 kg, from a local slaughterhouse (Macello Malvisi, Noceto, Parma, Italy) and transported to the laboratory in a freezer bag within 1 h and placed into cold (4 °C) sterile PBS supplemented with penicillin (100 IU/mL), streptomycin (100 µg/mL) and amphotericin B (2.5 μg/mL) [6]. A validated method [12] was performed to isolate adipose stromal cells (ASCs) from abdominal subcutaneous adipose tissue (about 5 g). Initially, samples were aseptically transferred in Petri dishes and minced (approximately 5 mm^3^ for each fragment). Thereafter, adipose tissue fragments were cultured as previously described [10,11]. The culture medium (CM) was represented by low glucose DMEM + GlutaMAX TMSupplement (GIBCO, Grand Island, NY, USA), supplemented with 10% fetal bovine serum (FBS), penicillin (100 IU/mL), streptomycin (100 IU/mL) and amphotericin B (2.5 μg/mL). The ASCs obtained were examined for the expression and the effects of Irisin (150 ng/mL) [4] on cell growth, redox status, and adipogenic differentiation.

### 2.2. ASC Growth

#### 2.2.1. Cell Proliferation

ELISA BrdU (Roche Diagnostic, Indianapolis, IN, USA), an immunological colorimetric assay, was employed to test the ASC proliferation. ASCs were seeded into 96-well plates (Sarstedt, Nümbrecht, Germany) (10^4^ cells/200 µL of CM), treated with Irisin (150 ng/mL) [4], and incubated for 48 h at 37 °C in a humidified atmosphere [13]. Subsequently, cells were incubated with 20 μL of BrdU for 24 h [11]. The cell number/well was determined from the resulting linear regression equation and used to correct the experimental data. The assay detection limit was 10^3^ cells/well, and the variation coefficient was less than 5%. Absorbance values were measured at 450 nm using a Victor Nivo spectrophotometer (Perkin Elmer, Groningen, The Netherlands).

#### 2.2.2. Metabolic Activity

ATP-lite (Packard Bioscience, Groningen, The Netherlands), a bioluminescent assay based on the production of light caused by the reaction of ATP with added luciferase and luciferin, was used to assess the ASC viability. The test was validated by plating different viable cell numbers (from 2.5 × 10^3^ to 4 × 10^6^/100 µL). The curve was repeated three times. The relationship between cell number and luminescence was linear (r = 0.95). An amount of 4 × 10^4^ cells/100 µL of CM was seeded in 96-well plates and treated with Irisin as detailed above. The assay was performed according to the instructions, and luminescence was finally recorded by the Victor Nivo luminometer [14]. 

### 2.3. ASC Redox Status Parameters

#### 2.3.1. ASCs Superoxide Anion (O_2_^−^) Production

The WST-1 (4-[3-(4-iodophenyl)-2-(4-nitrophenyl)-2H-5-tetrazolium]-1,3-benzene disulfonate) test (Roche, Mannheim, Germany) was set up to evaluate O_2_^−^ production [15,16,17]. An amount of 10^4^ viable cells/200 µL of CM was seeded in 96-well plates (Sarstedt, Nümbrecht, Germany), treated with Irisin as described above, and incubated for 48 h. In total, 20 μL of WST-1 was added to the cells during the last 4 h of incubation, and the absorbance was then determined using a Victor Nivo reader at a wavelength of 450 nm against 620 nm. 

#### 2.3.2. ASCs Nitric Oxide (NO) Production

NO production was assessed by measuring nitrite levels in the culture media by means of the Griess assay. An amount of 4 × 10^4^ viable cells/200 μL of CM was seeded in 96-well plates (Sarstedt, Nümbrecht, Germany) and treated with Irisin for 48 h as described above [18].

#### 2.3.3. ASCs Superoxide Dismutase (SOD) Activity

The SOD Assay Kit (Dojindo Molecular Technologies, Japan) was used to assess SOD activity. An amount of 4 × 10^4^ cells/200 μL of CM was seeded in 96-well plates (Sarstedt, Nümbrecht, Germany) and treated for 48 h with Irisin as detailed above. The colorimetric assay was performed as previously described [19].

#### 2.3.4. ASCs Non-Enzymatic Scavenging Activity

The ability of the antioxidant molecules to reduce ferric-tripyridyl-triazine (Fe^3+^TPTZ) to the ferrous form (Fe^2+^TPTZ) is the principle of the Ferric Reducing Activity of Plasma (FRAP) assay, a colorimetric method. Briefly, 4 × 10^4^ viable cells/200 μL of CM were seeded in 96-well plates (Sarstedt, Nümbrecht, Germany) and treated with Irisin for 48 h as described above. The test was performed according to Basini et al. [20].

### 2.4. ASC Adipogenic Differentiation

ASCs were seeded in CM and incubated for 48 h in a humidified atmosphere (37 °C, 5% CO_2_) to permit cell adhesion. Thereafter, adipogenic differentiation was induced by exposing the cells to the following treatments for 3 cycles of 4 days each [10,11]:

- Three days with an induction medium: culture medium supplemented with dexamethasone (DEX) 1 μM, insulin (INS) 1.7 μM, 3-iso-butyl-1-methylxanthine 0.5 mM, and indomethacin 250 μM;
- One day with a differentiation maintaining medium: culture medium supplemented with INS 1.7 μM.

Cell differentiation was evaluated by Oil Red O Staining to detect cytoplasmic lipid vacuoles as markers of adipogenesis. ASCs cultured in CM were employed as negative controls.

#### 2.4.1. Solubilization and Quantification of Oil Red O Production

To evaluate intracellular lipid accumulation, 2 × 10^4^ ASCs were seeded in 24 well-plates and subjected to the differentiation process in the presence or absence of Irisin as reported above. Based on the procedures previously detailed [8], the cells were stained with the Oil Red O staining solution for 30 min, and the absorbance at 540 nm was measured using a Victor Nivo spectrophotometer [10]. 

#### 2.4.2. Oil Red O Staining

A total of 5 × 10^4^ ASCs were seeded onto a cover slip in 6-well plates and subjected to the adipose differentiation process in the presence or absence of Irisin. After 20 days, the cover slips were fixed with formalin (4%) and stained with Oil Red O, prepared by mixing 6 parts of the Oil Red O stock solution with 4 parts of distilled water. The working solution was allowed to stand for 10 min and then was filtered using a Whatman filter n.1. The culture medium was removed, and the wells were washed twice with PBS. The cells were fixed in 10% buffered formalin for 30 min at room temperature. Next, the plate was rinsed with distilled water. The working solution of Oil Red O was pipetted to cover cell layers, and the dishes were slowly rotated to spread Oil Red O over the cells. After 5 min, the excess stain was cleared by adding 60% isopropanol, swirling, and then removing. Next, the cells were stained in Mayer haematoxylin for 3 min. Subsequently, images were taken by light microscopy (total magnification 10×) with a Nikon Eclipse E800 microscope (Nikon Instruments, Firenze, Italy) [11]. 

#### 2.4.3. Gene Expression Analysis

The gene expression of the Irisin precursor FNDC5 and adipogenic markers crucial for the function of adipose tissue, PPARγ and Leptin, was evaluated in the ASCs under basic conditions, and then assessed in ASCs subjected to adipogenic differentiation, treated or untreated with Irisin (150 ng/mL). The total RNA was extracted from adipose stromal cells pellets (7 × 10^5^ cells) using NucleoSpin RNA II (Macherey-Nagel GmbH, Duren, Germany). The cDNA was synthesized from 2 μg of RNA in reverse transcription reactions using the high-capacity cDNA reverse transcription kit (Applied Biosystem, Waltham, Massachusetts, USA), according to the manufacturer’s instructions. The mRNA levels were measured by real-time PCR using the Applied Biosystem StepOne™ Real-Time PCR System (Applied Biosystems, CA, USA), with a 20 µL reaction volume consisting of cDNA transcripts, PowerUp SYBR Green MasterMix (Life Technologies), and primer pairs (Eurofins Genomics, Ebersberg, Germany) shown in Table 1. Actin was used as a housekeeping gene. The thermocycler program was set as follows: initial pre-denaturation at 95 °C for 1 min, followed by 40 consecutive cycles consisting of denaturation at 95 °C, annealing of primers at 57 °C, elongation at 72 °C and finally, a further extension at 60 °C. 

The occurrence of the amplification was verified by 2.5% agarose gel electrophoresis (USB Corporation, Cleveland, USA) in TAE buffer (Tris base, glacial acetic acid, EDTA 0.5 M, pH 8), where the correct length of the amplicons was verified by comparison with Gene Ruler™ 100 bp DNA LaddeR markers (Fermentas, Waltham, MA, USA); as a negative control for all reactions, DEPC treated water was used instead of cDNA. The DNA patterns were displayed by UV lamp, and the images were captured with a PowerShot A610 Canon camera (Canon, Milan, Italy).

### 2.5. Statistical Analysis 

Each experiment was repeated 5 times. Adipose tissue was collected from the slaughterhouse from animals intended for human consumption to avoid the sacrifice of experimental animals. Each experimental treatment was performed with 6 replicates (wells) for each experiment. The Shapiro–Wilk test was used to confirm the normality of the data, which were analyzed without transformation. In all experiments, statistical differences were calculated by ANOVA using the Statgraphics Plus software (STC Inc., Rockville, MD, USA). When a significant difference of *p* < 0.05 was found, the means were submitted to the Scheffé F test.

## 3. Results

### 3.1. Expression of Irisin and the Effect on ASC Growth

**Figure 1:**
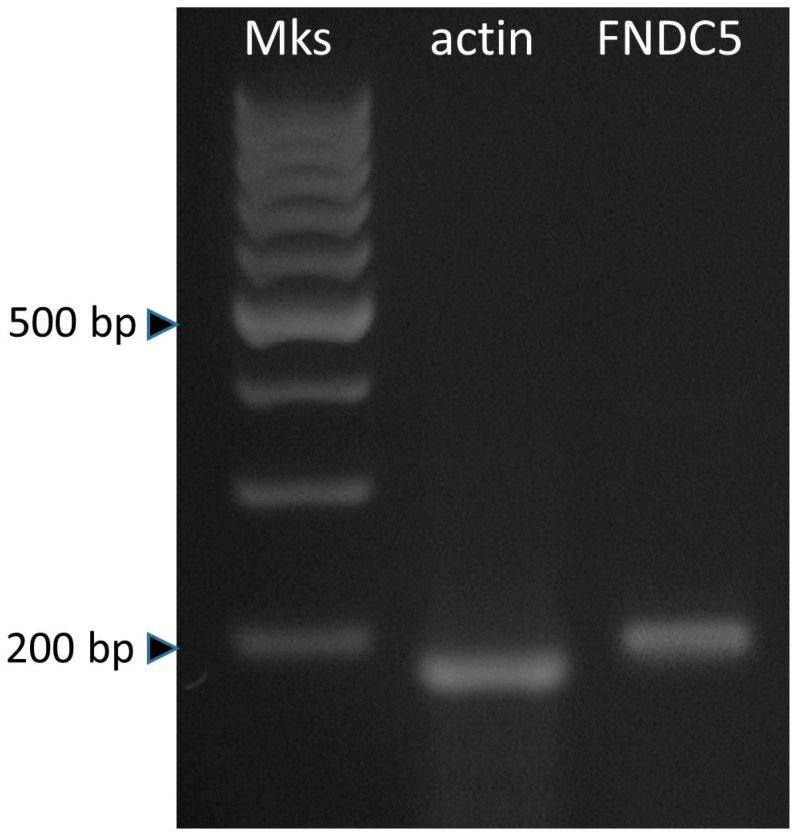
FNDC5 and actin expression in swine adipose stromal cells.

FNDC5 appeared to be expressed in adipose stromal cells (Figure 1).

**Figure 2:**
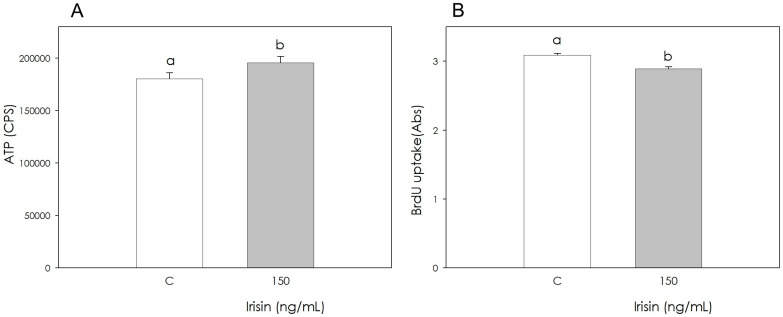
Effect of the treatment with Irisin (150 ng/mL) for 48 h on swine adipose stromal cell metabolic activity (**A**) measuring ATP production and proliferation (**B**) using 5-bromo-2′-deoxyuridine (BrdU) incorporation assay test. Data, expressed as count per second (CPS) (**A**) or milliAbsorbance (mAbs) units (**B**), represent the mean ± SEM of six replicates/treatment repeated in five different experiments. Different letters on the bars indicate a significant difference (*p* < 0.05) among treatments as calculated by ANOVA and the Scheffè’ F test.

The cellular metabolic activity, assessed by the production of ATP, was significantly stimulated (*p* < 0.05) by the presence of Irisin at a concentration of 150 ng/mL. A quantitative colorimetric assay based on the ability of bromodeoxyuridine (BrdU) to incorporate into DNA during the S phase was used to study the changes in cell proliferative activity. Irisin treatment induced a reduction in cell proliferation (*p* < 0.001) (Figure 2A,B).

### 3.2. Irisin Effects on Redox Status Parameters

**Figure 3:**
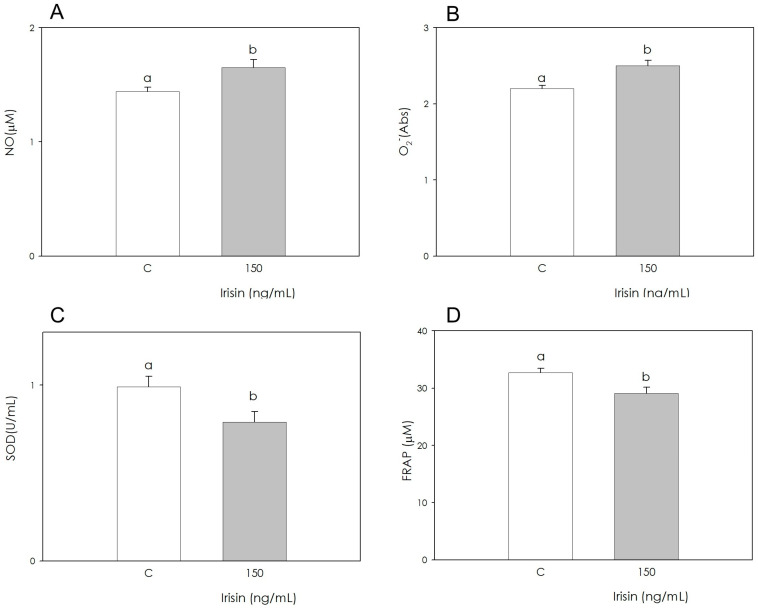
Effect of the treatment with Irisin (150 ng/mL) for 48 h on swine adipose stromal cell nitric oxide (NO; **A**), and superoxide anion (O_2_^−^; **B**) generation, superoxide dismutase activity (SOD; **C**) and non-enzymatic scavenging activity (FRAP; **D**). Data, expressed as µM (**A**,**D**), absorbance (Abs; **B**) or U/mL (**C)**, represent the mean ± SEM of six replicates/treatment repeated in five different experiments. Different letters on the bars indicate a significant difference (*p* < 0.05) among treatments as calculated by ANOVA and the Scheffè’ F test.

The measurement of NO levels was carried out using the Griess test. The results show that Irisin produced a stimulatory effect (*p* < 0.001) (Figure 3A). The quantitation of O_2_^−^ levels was achieved using WST-1 tetrazolium salt. Irisin significantly stimulated (*p* < 0.001) O_2_^−^ generation (Figure 3B). The antioxidant defense represented by SOD activity was reduced (*p* < 0.05) by Irisin (Figure 3C). The reducing power of cellular lysates was measured by means of a colorimetric assay; this technique made it possible to highlight that Irisin inhibited the antioxidant power of the sample (*p* < 0.001) (Figure 3D).

### 3.3. Changes in FNDC5, PPARγ and LEPTIN Gene Expression during Adipogenic Differentiation and Irisin Treatment

**Figure 4:**
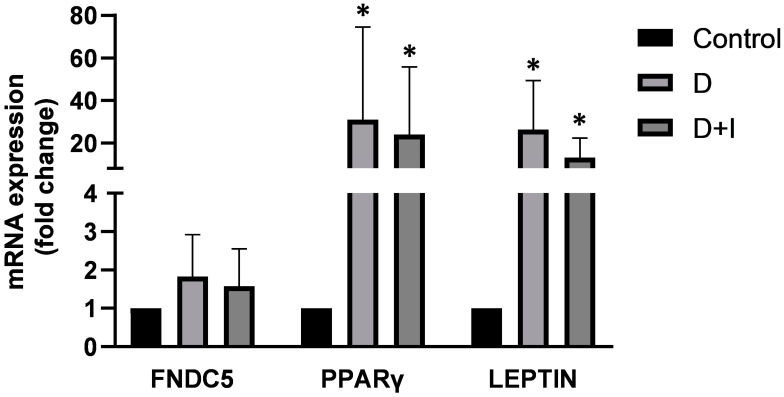
The figure shows the mRNA expression (fold change) of FNDC5, PPARγ and LEPTIN in two cell populations relative to the Control (ASCs pre-differentiated): ASCs differentiated into adipogenic lineage (D) and ASCs differentiated into adipogenic lineage and treated with 150 ng/µL of Irisin (D + I). The gene expression of PPARγ and LEPTIN was increased in D and D + I compared to the control (* *p* value ≤ 0.05), while no significant change was observed in FNDC5 expression between the three cell populations. Actin was used as a housekeeping gene.

The gene expression of FNDC5, PPARγ and LEPTIN was evaluated by Real-Time PCR (Figure 4) both in differentiated ASCs (D) and differentiated ASCs treated with Irisin (D + I). A significant increase in PPARγ and LEPTIN mRNA expression was observed in both differentiated cell populations (D and D + I) when compared to the control (undifferentiated ASCs), while no differences were assessed in the gene expression of FNDC5. Furthermore, Irisin treatment seemed to not influence the expression of these adipogenic markers at the concentration used (150 ng/mL).

### 3.4. Effect of Irisin on Adipogenic Differentiation

**Figure 5:**
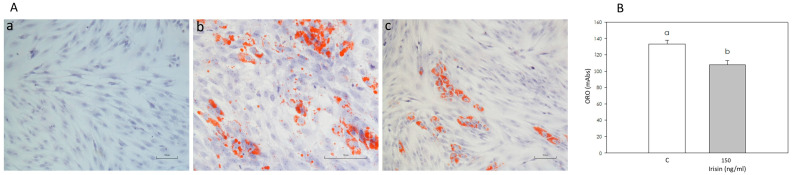
(**Panel A**): Optical microscope images (10×) of (**a**) control adipose stromal cells not differentiated, (**b**) adipose stromal cells undergoing adipogenic differentiation in the absence or (**c**) in the presence of Irisin 150 ng/mL, after Oil Red O staining. (**Panel B**): Spectrophotometric quantification (milliAbsorbance; mAbs) after Oil Red O staining of adipose stromal cells undergoing adipogenic differentiation (a) in the absence or (b) in the presence of Irisin (150 nL/mL) has to be changed in differentiation in the absence (C) or in the presence of Irisin (150 ng/mL).

Adipogenic differentiation of adipose stromal cells was achieved, as shown by the appearance of red lipid droplets in the differentiated cell cytoplasm (Figure 5(Ab)). The number of cells showing red lipid droplets was lower (*p* < 0.05) in cells induced in the presence of Irisin (Figure 5(Ac)) compared to the control. This result was confirmed by Oil Red O spectrophotometric quantification, which showed a decrease (*p* < 0.05) in the Irisin-treated sample absorbance (Figure 5B).

## 4. Discussion

Irisin, a myokine released from skeletal muscle into the bloodstream, has several targets, including adipose tissue. It has recently been shown that this peptide plays a crucial role in the “browning” process, by which white adipose tissue is converted into brown tissue, characterized by different morphofunctional characteristics [21]. A close correlation has also been documented between the circulating levels of Irisin and some cardiovascular diseases, including metabolic syndrome [22]. In particular, since evidence exists that patients affected by this condition have reduced levels of Irisin, this finding could represent a possible diagnostic marker. Other studies have reported that Irisin is produced in response to intense physical activity, thus suggesting that the benefits of physical exercise can be mediated, at least in part, by this hormone: this evidence could represent an interesting perspective on possible therapeutic strategies for dysmetabolic diseases such as diabetes and obesity [23]. Recent research has also revealed scenarios at the ovarian level [4] as well as on bone function regulation, in particular for the treatment of osteoporosis [24]. Furthermore, it has been observed that Irisin is also involved in learning and memory and has an anti-inflammatory effect on the CNS [25]. In particular, on the basis of the various recent studies, it is increasingly clear that this hormone may represent the potential link between fat metabolism, general metabolic activity, and physiological functions. In fact, Irisin is mainly released from subcutaneous white adipose tissue (WAT), and it plays a potential role in improving metabolic status as well as in curbing fat accumulation [26]. Adipose tissue, which hosts stromal mesenchymal cells that can be differentiated if adequately stimulated, is crucial for reproductive function. Although the cellular hierarchy of adipocyte progenitors is poorly understood, fat progenitor cells reside in the mesenchymal compartment of the tissue [7,8,27]. Recently, it has been demonstrated that Irisin can induce metabolic changes and the activation of specific pathways in adipose tissue ASCs (the effects of Irisin are mediated by binding to specific functional receptors (TRPC3, Irisin receptor)). These findings support our interest in exploring the role of this molecule on reproductive function [28,29]. Therefore, the purpose of this work was to investigate whether Irisin exerts effects on adipose tissue stromal cells (ASCs). Thus, we evaluated cell growth, taking into account both proliferation and metabolic activity and a series of parameters suggestive of the cell redox state. Moreover, we wanted to assess the potential Irisin effects on adipogenic differentiation. The experimental model chosen to perform the study was represented by the pig, which shares similarities in different anatomical structures with the human species and, at the same time, possesses a very high genetic homology. Moreover, pigs have very high birth rates and are easily reared, their dimensions are compatible with those of humans, and their use does not raise ethical problems that would arise, for example, with primates. Our first objective was to investigate the expression of the precursor of Irisin, FNDC5, in ASCs. The results we obtained highlighted the expression of FNDC5 in the samples of the ASCs analyzed. To our knowledge, current studies on ASCs are lacking, although the expression of FNDC5 has been documented in human muscle and in adipose tissue, in which different levels appear to be associated with obesity [30]. Varela-Rodríguez et al. [31] showed relatively high levels of expression in brown adipose tissue and modest expression in white adipose tissue deposits in rats. Komolka et al. [32] demonstrated the expression of FNDC5 in the subcutaneous adipose tissue in cattle. Currently, therefore, this study appears to be the first data demonstrating their expression in ASCs in the porcine model. Since these cells are able to differentiate into multiple cell lines, it is conceivable that this substance could modulate the differentiation process. In recent years increasing data support the hypothesis that these cells, or a subset of these cells, are indeed involved in the differentiation of adipocytes in fat tissue in vivo [7,8,27,28]. On these bases, the study of the ASCs secretome could be worthy of consideration. In fact, the interplay between ASCs, the local immune system and reproductive organs could play a role in reproductive function [5]. After the demonstration of expression in ASCs from porcine adipose tissue, we verified Irisin’s ability to influence the main functional aspects of these cells; its concentration was chosen on the basis of a previous work performed in our lab in porcine granulosa cells [4]. In particular, we studied the effects of Irisin on cell growth as well as a series of parameters characterizing the cell redox state. New DNA synthesis was used to evaluate proliferation, which was found to be inhibited by treatment with Irisin; on the contrary, ATP production was significantly stimulated. Therefore, the data suggest a negative effect on cell proliferation and a positive interference with metabolic activity. In agreement with our data, Xie et al. [33] demonstrated that Irisin inhibits cell proliferation in rat cardiomyoblasts. We obtained similar results in a previous study performed in porcine granulosa cells [4]. Additionally, Liu et al. [34] documented that Irisin can inhibit the growth of pancreatic cancer cells. On the other hand, Irisin displayed a stimulatory effect on human endothelial cells [35] as well as on rat pancreatic cell proliferation [36]. Mazur-Bialy [37] demonstrated an increased proliferative activity induced by Irisin in mouse macrophages. These conflicting data could be due to the different concentrations of Irisin used, the cell type specificity, as well as the culture and analytical methods used. The data we obtained in our work led us to hypothesize the role of Irisin in controlling the development of adipose tissue through the inhibition of ASC proliferation. Moreover, Irisin’s effects on metabolic activity seem contrasting. A stimulatory action was documented in rat cardiomyocytes [38] and cells [39], while Kuloglu et al. [40] reported that Irisin does not induce the production of ATP in mouse cardiomyocytes. With regard to this aspect, the conflicting data may be due to the different concentrations used. Since metabolic activity is closely related to the production of free radicals and the synthesis of ATP through the respiratory chain and involves the consumption of molecular oxygen and the release of ROS, an increased activity could cause oxidative stress. Overall, since it appears likely that Irisin can alter the cellular redox state, it seems interesting to evaluate a series of parameters that characterize this aspect in porcine ASCs. The balance between reactive species and antioxidants is essential for proper cellular function since the increase in free radical levels could exceed the capacity of an individual’s antioxidant defenses, placing one at risk of oxidative stress. Therefore, in the present study, we also examined the potential effect of Irisin on radical species and antioxidants. In this work, we have shown that Irisin treatment produced a stimulatory effect on NO and O_2_**^−^** production. In previous work carried out in our laboratory on porcine granulosa cells, we obtained contrasting results since the treatment with Irisin significantly inhibited NO production, while no changes in O_2_**^−^** production were found [4]. Fu et al. [41] documented a stimulation of NO production resulting from an increase of eNOS activity in rat endothelial cells, and Deng et al. [42] reported a dose-dependent increase in NO release in human umbilical cord cells (HUVEC) treated with Irisin. We also evaluated the non-enzymatic antioxidant power, which was decreased by Irisin; additionally, the specific enzymatic activity of SOD was significantly inhibited. Conflicting evidence emerged from the study conducted by Wang et al. [43] on mouse cardioblasts, where an increase in SOD-1 activity was observed after treatment with Irisin, suggesting an antioxidant role of myokine. On the whole, our results show that Irisin increases the synthesis of NO and O_2_**^−^** and, at the same time, inhibits the antioxidant power. ROS, produced during physical exercise, are recognized as responsible for the positive regulation of gene transcription, which causes an increase in mitochondrial biogenesis and, consequently, an increase in the energy potentially developed by the muscle, thus gaining greater oxidizing capacity; therefore, during work, its ability to produce and use energy increases. Some studies have shown that physical exercise and the mechanical stimulation of muscle contractions potentiate the production of Irisin in mice and humans, increasing energy expenditure [1,44]. This hormone is involved in multiple functions, including “browning”, which improves the oxidation of fatty acids. Given the evidence that exercise also enhances the production of NO, which, in addition to causing vasodilation, improves blood flow and microcirculation and intervenes in energy metabolism, it is possible that the myokine, also involved in increasing the concentration of ATP, plays a role in the mediating of these effects. Therefore, it could be hypothesized that this molecule may be involved in the increase of free radicals in muscle and adipose tissue. Moreover, the augmented NO production in adipocytes resulting from physical activity has been shown to have a positive action in stimulating lipolysis in adipose tissue as well as in decreasing the fat accumulation in the abdominal subcutaneous adipose tissue [45]. These data suggest that the use of Irisin could represent a therapeutic strategy in the prevention of obesity. In addition, we also evaluated the effect on cellular differentiation of ASCs towards adipocytes by Oil Red O staining. As previously reported, Irisin can interfere with adipose tissue differentiation and metabolism. A metabolic and endocrine role for fat tissue has been proposed on the reproductive axis [46]. Thus, exploring Irisin’s role in ASCs function could be informative to evaluate its eventual effects on reproductive function. The results we obtained indicate that Irisin inhibits differentiation. A study by Li et al. [47], in accordance with our data, demonstrates a similar effect in human visceral adipocytes, and Zhang et al. [48] documented a reduced adipogenic differentiation in human perirenal adipose tissue. On the contrary, Luo et al., 2020 [49] reported a stimulatory action on osteoblast differentiation.

In conclusion, the collected data suggest a local role of Irisin in the modulation of adipose tissue function.

## Figures and Tables

**Table 1:** Primers used for real-time PCR.

Gene	Accession Number	Primer Sequences	Reference
FNDC5	XM_02109583.1	F: 5′-TGCAGGCCATCTCCATTCAG-3′ R: 5′-ATATTGGCGGCAGAAGAGGG-3′	[4]
Actin	XM_003124280	F: 5′-ATGGATGACGATATTGCTGC-3′ R: 5′-CCCACGTAGGAGTCCTTCTG-3′	[4]
PPARγ	XM_013981982.1	F:5′-GCCCTTCACCACTGTTGATT-3′ R:5′GAGTTGGAAGGCTCTTCGTG-3′	[4]
Leptin	NM_213840.1	F:5′-TGGCCCTATCTGTCCTACG-3′ R:5′-TTTCTGGAAGGCAGACTGGT-3′	[10]

## Data Availability

All data generated or analyzed during this study are included in this published article. Original data will be available upon reasonable request.
